# In search of solutions to grapevine trunk diseases through “crowd-sourced” science

**DOI:** 10.3389/fpls.2013.00394

**Published:** 2013-10-02

**Authors:** Karen L. Block, Philippe E. Rolshausen, Dario Cantu

**Affiliations:** ^1^Department of Viticulture and Enology, University of California DavisCA, USA; ^2^Department of Botany & Plant Sciences, University of California RiversideCA, USA

**Keywords:** grapevine trunk diseases, eutypa dieback, pathogenomics, crowdsourcing, data-share, botryosphaeria dieback, esca dieback

## Introduction

Trunk diseases create tremendous problems for the viticulture industry causing significant economical losses due to reduced yields, increased crop management costs for cultural and chemical preventive measures, and shortened life span of the vines (Munkvold et al., 1994; Bertsch et al., 2013). No cures are currently available and the diseased wood is typically removed to limit the spread of infection to other permanent structures of the vine. Knowledge of the mechanisms responsible for the development of these diseases is still limited. Recent advancements in genome sequencing technologies (so-called, Next Generation Sequencing) allow us to quickly catalog the entire repertoire of virulence functions of a plant pathogen, a rapid acquisition of a huge amount of information for organisms that were previously uncharacterized. We have recently released the draft genome sequences of pathogens associated with three major trunk diseases of grapevine: esca dieback (*Phaeoacremonium aleophilum*), botryosphaeria dieback (*Neofusicoccum parvum*), and eutypa dieback (*Eutypa lata*). All data, including the raw sequencing data, have been deposited in public databases (GenBank WGS and SRA archives) and are also hosted on the Cantu Lab website (http://cantulab.github.com). The data is freely available to anybody with no restrictions. Sequencing, assembly and preliminary genomic analyses are described in the journal *Genome Announcements* published by the American Society of Microbiology (Blanco-Ulate et al., 2013a,b,c). These one-page reports provide scientists that want to use the genomic information with details on the pathogen strains sequenced and how the sequencing and assembly were performed. With the exception of the genome of *Botrytis cinerea* (Amselem et al., 2011) the fungal agent of bunch rot, to our knowledge these are the first genomes of fungal grape pathogens publicly available. Updates to the current genome versions will be readily shared through the Cantu lab website hosted by GitHub (http://github.com), which will also provide a centralized repository for bioinformatic scripts and pipelines used in the genome analyses. The availability of newer versions will be communicated through Twitter (@CantuLab), or any social media that is widely used. By sharing these novel genomic sequences through rapid public release, we hope to stimulate research/scientific crowdsourcing to understand how these destructive pathogens cause disease, reducing grapevine yield and lifespan. This aggregation of talent and complementary expertise, with no need to initiate formal collaborations, will significantly reduce the time needed to solve these critical viticulture problems. We also hope that our openness will help promote the more collaborative model of scientific approach.

## Grapevine trunk disease pathogens

The first pathogen we sequenced was a strain of the fungus *Eutypa lata*. This particular strain was isolated from diseased vines in Fresno County (California) in 2011. *E. lata* had long been thought to be the primary cause of grapevine dead-arm and dieback symptoms until other pathogenic fungi were also found to be associated with these disease symptoms. Symptoms of dieback appear several years after *E. lata* infection on older vines (8–10 years) when, particularly in spring, small chlorotic leaves with shortened internodes appear on stunted shoots (Carter, 1991; Péros and Berger, 1994). At the infected spur positions many flowers often dry before blooming, while many of the successfully fertilized flowers develop into small and sparse berries. Grape cultivars show differences in their susceptibilities to *E. lata* (Rolshausen et al., 2008), but no resistant cultivars or completely effective management practices are currently available. *E. lata* enters the host through pruning or grafting wounds, colonizes the vascular tissues, and gradually kills the plant (Carter, 1991; Péros and Berger, 1994).

The second pathogen we sequenced was a strain of the fungus *Neofusicoccum parvum* isolated from infected grapevines in Riverside County in 2011. Several species in the *Botryosphaeriaceae* family, including *N. parvum* are opportunistic pathogens of grapevines causing the so-called *botryosphaeria* dieback (Urbez-Torres, 2011). *N. parvum* penetrates grapevines through pruning and grafting wounds and colonizes the host tissues, causing shoot dieback, cane bleaching, bud necrosis, and graft failure. Wedge-shaped necrosis in the vascular tissues of spurs, cordons, and trunks are typical symptoms of botryosphaeria dieback (Úrbez-Torres and Gubler, 2009, 2011).

The third pathogen sequenced in this series, was a strain of the fungus *Phaeoacremonium aleophilum*, isolated in Fresno County (California) in 2011. This pathogen is associated with the esca dieback complex (Surico et al., 2008; Bertsch et al., 2013), which refers to five syndromes: brown wood-streaking, petri disease, young esca, esca, and esca proper (Surico et al., 2008), which are caused by *P. aleophilum* and other fungal species. Disease symptoms internal to the vine include, wood discoloration, streaking, and vascular necrosis. External symptoms include, leaf chlorosis and necrotic stripes, berry black spots, decline in vigor and yield, and in severe cases plant death (Mugnai et al., 1999; Bertsch et al., 2013). Species associated with esca dieback and botryosphaeria dieback have also been observed to survive within the plant tissues as latent endophytes becoming pathogenic only under certain environmental and physiological conditions (Mugnai et al., 1999; Slippers and Wingfield, 2007).

Pruning wounds are the main entry point of these fungi. Because there is no curative treatment for these diseases once they colonize the vine, preventative treatment with fungicide application on wound surfaces is the only management strategy. However, the incubation period between the initial colonization of the vine and the appearance of the first symptoms (years in the case of *E. lata*) deceive growers into a false sense of security and thus they often do not spray because vines look healthy at least in the first years following the establishment of the vineyard. Once diseases are established and symptoms are expressed, it is too late. In addition, the labor costs associated with fungicide application (because often done by hand on every single wound following pruning) often prohibits the implementation of a management strategy at a large scale. Too often, growers do nothing to protect their vines hoping they will not be too severely impacted by these diseases (Rolshausen et al., 2010).

The research focus of the scientific community has for years been on finding preventative fungicide treatments that are effective. Also, there has been much work done on the identification of the causal agents of trunk diseases in various wine growing regions. *E. lata*, *N. parvum* and *P. aleophilum* are major cosmopolitan pathogens, but many others with a more restricted geographical range have recently been identified. However, the long incubation time required for these pathogens to express symptoms does not make them an easy model system to study. The turnaround from setting up the experiment to publication takes months, sometimes years, and is not suited for the academic system requirements of high volume publications. In other words, these pathosystems are not attractive for young faculty trying to get tenure. For these reasons, the research on trunk diseases has been limited in scope. The sequencing of genomes and crowdsourcing provide tools for new research avenues that will help find alternative management strategies to the one currently in place.

The effective colonization of the vine with killing and rotting of the tissues appears to depend on the capability of these organisms to produce toxic metabolites and enzymes that decompose the plant cell walls. By computational prediction and annotation, we identified genes in the genomes of the three species that may be playing important roles in killing and rotting grapevine tissues, but where do we go from here? Genomicists should continue sequencing more pathogen isolates and species, because more sequence diversity allows for more information on the evolution of pathogenicity and host specificity, all necessary information in order to develop broad and long term solutions to the problem. But this is not enough. We need a broader pool of experts if we really want to figure out the mechanisms in order to eradicate diseases. So we need pathologists, epidemiologists, biochemists and geneticists to look at toxin biosynthesis, cell wall degradation, new methods to screen grape germplasm for tolerance to trunk diseases, and improved diagnostic tools for early detection of the pathogens in asymptomatic vines, etc.

## Crowdsourcing

One solution that is rapidly gaining popularity in science is crowdsourcing. In a scientific context, crowdsourcing is the open decentralization of the experimental and analytical processes to involve as many participants as possible to solve a scientific question (Cooper et al., 2010) or a current problem (MacLean et al., 2013). This approach radically enlarges the pool of potential scientific contributors to a project, which, by broadening the spectrum of expertise, increases the chances of solving a particular problem, such as the eradication of grapevine trunk diseases. Because the availability of genomic information is often a critical bottleneck to further experimentation, the early release of the genome of an organism is the necessary entry point to crowdsourcing. As an example, since the grapevine PN40024 genome sequence was released in 2007, the original *Nature* paper has been cited over 900 times (The French-Italian Public Consortium for Grapevine Genome Characterization, 2007). The exponential increase in the number of scientific papers in this field after the public release of the grapevine genome provides evidence of how genomic resources can enable future experimentation. The improvement of sequencing and genotyping technologies along with computational tools have provided us with an unprecedented capability of rapidly generating genomic information for any organism of interest. While the sequence of a single pathogen isolate is not sufficient to understand how organisms evolve processes to overcome host immunity and spread to multiple plant species, it provides a nearly complete catalog of virulence capabilities, information that can be used by a broad spectrum of scientists to understand how these trunk pathogens infect and kill grapevines helping to develop improved diagnostic and disease control tools. More importantly, such an approach of open engagement with respect to research will also allow live peer-review, with independent researchers contributing, reviewing and analyzing the data, leading to an improvement in the quality of the data. Thus, the early release of genome sequences benefits from a distributed peer-review and improvement of the data and, at the same time, benefits the community by providing critical information.

Crowdsourcing has already been successfully applied in several fields. A remarkable example is Foldit, a community-based effort that uses “human intelligence” to optimize protein structure prediction (Cooper et al., 2010). Despite the success of Foldit, crowdsourcing has not experienced wide-spread application in the life sciences, mostly because of the current methods of evaluation of scientific impact and academic performance that are based on publication in peer-reviewed journals and journal citation analyses (Salisbury, 2013). In this traditional system, scientists benefit from keeping data secret, including genomic sequences, until formal acceptance of their publication in a peer-reviewed journal, which may happen years after the data are produced. This data secrecy is necessary to be able to publish in a high-impact journal, a critical product for career advancement in academia. Despite the clear value and possibly broad impact of early release of the genome sequence of an organism of common interest, a data set *per se* is not currently formally evaluated as a scientific product in the academic context. The introduction of journals, such as *Genome Announcements* (http://genomea.asm.org), represents a step forward to promote the early release of genome sequences. *Genome Announcements* publishes short reports that describe genome sequences that are available in public databases, which not only provide valuable basic technical information to the reader, but also a citation from anyone that uses the data. Additionally, the scientific community is rapidly acquiring new ways to evaluate the impact of published works that go beyond the traditional bibliographic ranking based on academic journal citation analysis. These new forms of measuring scientific impact, such as Altmetrics, use the number of Twitter tweets, Facebook shares, and saves on Delicious and Mendeley to measure the article's popularity in real time, tracking how fast it spreads throughout the community. While traditional peer-review happens before publication, internet and social media provide an avenue for continued peer evaluation, correction, and improvement after publication.

The introduction of new journals, the spread of open access publication policies to major institutions, such as the University of California system (http://senate.universityofcalifornia.edu/open_access_press_release_2013.pdf), new forms of impact evaluation, and frameworks for collaborative analysis, such as Github and Synapse (http://sagebase.org/synapse/), hold the promise to facilitate the quick and open flow of data to accelerate scientific discoveries while rewarding those who generate data and provide open-access to critical information that benefits the community, which ultimately improves the scientific product through temporally extended and broader peer-revision. Just this year, MacLean et al. put out a call for crowdsourcing the genomic analyses of ash and ash dieback (MacLean et al., 2013). We believe this is the beginning of the stampede and welcome people with the appropriate expertise to help analyze the genomic information we have shared, contributing to solve one of the most pressing problems of the grape industry.
